# Recombinant SARS-CoV-2 Spike Protein Mediates Glycolytic and Inflammatory Activation in Human Monocytes

**DOI:** 10.1093/geroni/igaa057.3493

**Published:** 2020-12-16

**Authors:** Brandt Pence

**Affiliations:** University of Memphis, Memphis, Tennessee, United States

## Abstract

Severe acute respiratory syndrome coronavirus-2 (SARS-CoV-2) causes coronavirus disease 2019 (COVID-19) in part through cytokine storm. Metabolic reprogramming in immune cells mediates inflammation, and recent evidence suggests SARS-CoV-2 activates glycolysis in monocytes to facilitate cytokine production. In this study I investigated the ability of the spike protein (subunit 1) from SARS-CoV-2 to cause glycolytic reprogramming and inflammatory activation in isolated human monocytes. Primary human monocytes were isolated from healthy young donors (N=4) by immunomagnetic negative selection and stimulated with recombinant SARS-CoV-2 spike protein subunit 1 (rS1) for 6 hr. Glycolysis was monitored by assessing extracellular acidification using a Seahorse assay. Supernatants and cell lysates were subsequently processed for gene and protein expression assays by qPCR and ELISA respectively. Treatment of monocytes with rS1 at 10 nM and 30 nM led to significant upregulation of glycolysis, as well as a substantial increase in gene and protein expression of interleukin-6. Mouse bone marrow-derived macrophages did not display enhanced glycolysis when stimulated with rS1, suggesting a specific interaction of the protein with the ACE2 receptor, rather than a general inflammatory response caused by contamination with endotoxin or similar. Glycolytic activation in monocytes in response to rS1 suggests that immunometabolic modulators, including common geroprotectors such as rapamycin and metformin, may have efficacy in treating COVID-19.

